# Editorial

**Published:** 1871-05

**Authors:** 


### Editorial.

***Our Indiana Friend***

Wants to know what the Senior Editor's private notions are about the use of opium in metro-peritonitis, etc., etc. The Senior Editor has not the slightest objections to advance them publicly or privately.

With regard to the case referred to, he does not believe it was one of metro-peritonitis at all, and has strong suspicions that the same case has figured in a published report of opium-poisoning cured by belladonna. But these minor blemishes may be put up with.

When any physician uses alcoholic stimulants, veratrum viride, lobelia, capsicum, opium, quinine, oil of sassafras, hydrastis, or any other member of the materia medica, large or small, as a specific, or under the governance of some crude theory of its *modus operandi*, without positive reference to the essential relation of the medicine to the pathology of the particular case, he practices a bald empiricism which no repetition of successful results can entitle to a higher designation.

We have in our time opposed excessive blood-letting (Paine-ism), excessive stimulation (Toddism), excessive quininism, excessive sedation, excessive narcotism, etc., etc., with equal zeal and confidence of sound philosophy, as when laughing at the puerilities of homœopathy, or cautioning against the noisy claims of advertising knaves.

Let all things be done decently and in order. Opium is a priceless boon to suffering humanity. In the hands of the judicious physician it is capable of achieving the highest triumphs of the art—in the hands of the beetle-headed believer in its being "good for metro-peritonitis," or any other disease, it is capable of peopling, with admirable dispatch, the most capacious graveyards.

No remedy with which we are acquainted has a *single* physiological, or pathological, effect in its application to the human body under its infinitely varying conditions.

One man's *medicine* is another man's poison. Nay, it may be poison to the same man at a different time. What is "good for" any given change of the normal order in the system, is that which will restore that normal order—be it the order of function or of those integral changes upon which the manifestation of function depends.

All sedatives are capable according to their application of proving stimulant. All stimulants are capable of producing sedation. The most familiar illustration of this is in the physiological effects of cold and heat. To the sense totally dissimilar, yet in fact capable each of producing sedation, each of producing stimulation.

This is a general law, necessarily including all influences, whether of matter or force, operating on the human body.

Opium well illustrates this law. According to its application it is capable of increasing the amount of tissue change (stimulation), or of diminishing the same (sedation.)

Were this all, we could, perhaps, lay down pretty close rules for its administration. But, unfortunately, this touches only the edge of the matter. The body is an aggregate of dissimilar parts, each largely dependent on the perfection of operation of every one of the rest, for its own right action.

How it should happen that any single, so-called, medicine should agree with more than one person, is more to be wondered at than the opposite. Opium in any form, or any of its derivatives, causes the writer unmixed misery, but some of his professional friends are transported by its influence to an Elysium of happiness with clear consciences and breaths void of whisky.

There was a dapper little Frenchman who crossed Niagara, again and again, on a tight rope, pirouetting and capering, meanwhile, to the admiration of all beholders. Safe enough for Monsieur Blondin who knew the ropes and the balance, but tolerably unsafe, we opine, for the majority of the bystanders.

So, when Messieurs Blondins—Paine with his lancet, Todd with his brandy, Clarke with his opium, Norwood with his veratrum viride, Lente with his calomel, *et alii*, *et al.*, play fantastic tricks before high heaven, we will religiously presume it is safe enough for them—but for the rest,—*Timeo Danaos!*

The tools for those who know how to use them, said the First Napoleon. If you know your tool perfectly, even though it be opium, or that other concoction "nameless forever here," and if you know exactly what change is desirable in the diseased body, why, "Lay on, Macduff," and M.D—d be he who first cries "hold, enough"—that is, if your patient holds enough.

If our opium Blondin knows exactly when he not only lessens the unnecessary tissue metamorphosis in the affected organ, but dangerously diminishes it in sound organs, he will probably have sense enough to stop it. The bystander, with his head full of crotchets instead of sense, will never see this point, but will crowd the opium.

Control pain? Of course. Retard destructive metamorphosis? Yea. By opium? Most certainly, if possible. Regardless of dose? Yes, but *not* regardless of common sense. Dear Brethren—do continue the good work of mixing your medicines with brains, before you force them down the throats of the unfortunates committed to your charge.

***Recurring to First Principles.***

In a case of delirium tremens (diagnosed, by the way, as cerebritis) in a well known eleemosynary institution of this city, the *interne* caught a live chicken, split it open, and applied it, still warm, to the patient's abdomen as a poultice. Both the patient and the chicken died—the interne—he of the Washingtonian Home—recovered. 1871. Mark, time!

***Across the Continent.***

At the present writing, many of our brethren, anxious for the elevation of the profession, are bravely fronting the dangers of the Pullman Palace Cars, bad champagne, and worse whisky. Let them go, surrounded by the halo of a righteous cause, and protected from harm by the viewless legion of our best wishes. When they arrive at their destination and enter upon the discharge of their onerous but honorable duties, they will still be held up on the broad shoulders of our devotional petitions.

We trust they will steadfastly determine to make the association a scientific society, and not a mere trades-union or professional criminal court.

We trust they will let alone the work of the medical colleges, which is emphatically none of their business, and endeavor to have their membership keep out of the way of the majority of the medical students.

We trust that, individually, they will receive, "mark, learn and inwardly digest" the truth, that the great reason why the medical profession fall below their appropriate position in public estimation, is not because the colleges do not furnish pabulum enough for the neophytes, but because both the neophytes and full grown doctors are wanting in that primary education and generous culture, without which the most fertile soil of mind is more productive of weeds, of false and distorted views, of unphilosophical assumptions and absurd practices, than of that clear and accurate thought which makes the medical man strong in himself, and a power in community which will everywhere be respected. Educate from the beginning. Look out for the roots, and the branches will take care of themselves.

---

***Medical and Surgical Memoirs.*** By JOSEPH JONES, M.D., Professor of Chemistry, Medical Department, University of Louisiana. (In Preparation.)

This work will embrace the investigations of fifteen years, into the Causes, Geographical Distribution, Natural History and Treatment of Intermittent, Remittent and Congestive Malarial Fevers, Yellow Fever, Typhoid and Typhus Fevers, Small Pox, Spurious Vaccination, Measles, Pneumonia, Diarrhœa, Dysentery, Scurvy, Tetanus, Cerebro-Spinal-Meningitis, Diseases supervening upon gun shot wounds, Pyœmia, Hospital Gangrene, Erysipelas, etc., etc.

The results of the investigation of the diseases of the Confederate Army, during the American Civil War, 1861–5, will occupy a prominent portion of the work.

These investigations have been prosecuted unremittingly during the past fifteen years; and the author proposes to lay the results before the medical profession, as soon as a sufficient number of subscribers have been obtained.

Physicians and others desiring to become subscribers, will please forward their names; and those receiving the circular are respectfully requested to call the attention of their friends, and also of the County and State Medical Societies to the proposed work.

Address—

JOSEPH JONES, M.D.,

***Glass Box 1542, New Orleans.***

